# Development of osmotic vacuolization of proximal tubular epithelial cells following treatment with sodium-glucose transport protein 2 inhibitors in type II diabetes mellitus patients-3 case reports

**DOI:** 10.1007/s13730-021-00609-7

**Published:** 2021-05-22

**Authors:** Shun Watanabe, Naoki Sawa, Hiroki Mizuno, Masayuki Yamanouchi, Tatsuya Suwabe, Junichi Hoshino, Keiichi Kinowaki, Kenichi Ohashi, Takeshi Fujii, Yutaka Yamaguchi, Yoshifumi Ubara

**Affiliations:** 1 Nephrology Center, Toranomon Hospital Kanagawa, 1-3-1, Takatsu, Kawasaki, Kanagawa 212-0015 Japan; 2 Department of Pathology, Toranomon Hospital, Tokyo, Japan; 3 Department of Pathology, Yokohama City University Hospital Graduate School of Medicine, Kanagawa, Japan; 4 Okinaka Memorial Institute for Medical Research, Toranomon Hospital, Tokyo, Japan; 5 Yamaguchi’s Pathology Laboratory, Chiba, Japan

**Keywords:** Acute kidney injury (AKI), Isometric vacuolization, Osmotic vacuolization, Proximal tubules, Sodium-glucose transport protein 2 (SGLT2), Type II diabetes mellitus (T2DM)

## Abstract

We encountered 3 cases of acute kidney injury that occurred after treatment with a SGLT2 inhibitor. In case 1, serum creatinine increased from 1.65 to 3.0 mg/dL, in case 2, serum creatinine increased from 1.03 to 1.21 mg/dL, and in case 3, serum creatinine increased from 0.8 to 1.1 mg/dL. Renal biopsy showed isometric vacuolization on tubules, that was completely negative for Periodic acid-Schiff (PAS) stain in case 1, and was partially negative for PAS stain in case 2 and 3, consistent with osmotic vacuolization. Immunohistochemical analysis showed positive staining for CD138 and CD10 indicating the proximal tubules in the vacuolar lesions. 3 patients were obese with body mass index of more than 30, and showed an increase in serum renin. In conclusion, in type II diabetes mellitus (T2DM), individuals that remain within their standard weight range, SGLT2 inhibitor treatment does not result in osmotic vacuolization of proximal tubular epithelial cells and AKI. However, treatment with a SGLT2 inhibitor may cause damage of the proximal tubules resulting in AKI in T2DM individuals who do not remain within their standard weight range, due to an overdose lavage of sugar in the urine and dehydration.

## Introduction

Sodium-glucose transport protein 2 (SGLT2) inhibitors are medications that inhibit the reabsorption of glucose in the kidney, and therefore lower blood sugar levels, by inhibiting SGLT2. Consequently, SGLT2 inhibitors are used in the treatment of type II diabetes mellitus (T2DM). Treatment with SGLT2 inhibitors in T2DM patients has been reported to assist in blood sugar control, maintain an individuals’ body weight, along with systolic and diastolic blood pressure [1, 2]. The adverse events observed with SGLT2 inhibitors has been considered to be minimal and non-specific. We experienced 3 cases developed AKI and osmotic vacuolization of proximal tubular epithelial cells after treatment with a SGLT2 inhibitor in T2DM.

## Patients

Among 42 T2DM patients from 2015 to 2019 who received renal biopsy for renal dysfunction or proteinuria, we encountered three patients with definite isometric vacuolization on proximal tubules. Out of these 42 patients, we noticed that SGLT2 inhibitor was administered for these only three patients. We investigated whether a close relation between SGLT2 inhibitor and isometric vacuolization on proximal tubules exists or not.

The study procedures were in accordance with the Declaration of Helsinki. The study and its revisions were approved by the local research ethics board (approval number: 1633).

## Renal histology

Renal biopsy specimens were processed as previously described [3] for light microscopy (LM), immunofluorescence microscopy (IF), and electron microscopy (EM). All biopsy specimens were reviewed by 4 pathologists. Immunohistochemistry (IHC) analysis for CD138, CD10, E-cadherin, epithelial membrane antigen (EMA), cytokeratin 7 (CK7), and cytokeratin 34-beta E12 (CK34βE12) was also performed to examine a localization on tubules of vacuolization in the three patients diagnosed with osmotic vacuolization, as previously described [4, 5] (Table.1). Clinical characteristics of three patients is summarized in Table 2.

**Table 1:** Staining for common antigens of tubules

		Case 1	Case 2	Case 3
CD138	Proximal convoluted tubule	+	+	+
CD10	Proximal convoluted tubule and proximal straight renal tubule	+	+	+
E-cadherin	Henle loop, thick ascending limb, macula densa, distal convoluted tubule and collecting duct	−	−	−
EMA	Henle loop, thick ascending limb, macula densa, distal convoluted tubule and collecting duct	−	−	−
CK7	Henle loop, thick ascending limb and distal convoluted tubule	−	−	−
CK34βE12	Collecting duct	−	−	−

(+) shows positive staining, and (−) shows negative staining for EMA, epithelial membrane antigen; CK7, cytokeratin 7; CK34βE12, cytokeratin 34 beta E12

**Table 2:** Laboratory findings on patient admission

		Normal range	Case.1	Case.2	Case.3
Age	(year-old)		41	55	52
Weight (before)			84	88	80
Weight (on admissin)	(kg)		93	95.8	82
Hight	(cm)		162	169	164.5
BMI			35.4366712	33.5422429	30.3027503
Blood pressure	(mmHJg)		128/76	130/75	122/74
Duration of diabetes	(years)		7	5	17
type of SGLT2i			Dapagliflozin	Dapagliflozin	Dapagliflozin
Prescription period of SGLT2i	(month)		19	24	5
OHA (the others)	Biguanide		○		○
	DPP-4 inhibitor		○	○	○
	SU		○		○
	Glinide				
	Pioglitazone				○
	αGI				
Hypertension			○	○	○
Dyslipidemia			○	○	○
Hyperuricemia			○	○	
Blood test					
HbA1c	(%)		6.1	6.5	7.4
Blood sugar	(mg/dl)		74	86	108
TP	(g/dl)		7.7	6.7	8.0
Alb	(g/dl)		4.9	4	5.0
AST	(IU/l)		15	15	29
ALT	(IU/l)		24	20	37
LD	(IU/l)		138	133	175
ALP	(IU/l)		166	137	213
γGTP	(IU/l)		36	25	296
UN	(mg/dl)		28	15	16
Cre (before)	(mg/dL)		1.65	1	0.8
Cre (after)	(mg/dl)		3.02	1.2	1.1
eGFR (before)	(ml/min/m2)		36.7	59.5	79.7
eGFR (after)	(ml/min/m^2^)		19.9	55.3	74.6
UA	(mg/dl)		5.4	6	8.7
Na	(mEq/l)		141	144	138
K	(mEq/l)		3.6	4.3	4.2
Cl	(mEq/l)		100	109	100
Ca	(mg/dl)		9.4	9.4	9.9
IP	(mg/dl)		4.1	3.2	3
T-Bil	(mg/dl)		1.9	0.9	0.8
D-Bil	(mg/dl)		0.2		
TG	(mg/dl)		263	170	545
T-Chol	(mg/dl)		139	149	240
HDL-Chol	(mg/dl)		36	37	44
LDL-Chol	(mg/dl)		74	78	115
CRP	(mg/dl)		0.1	0.1	0.05
WBC	(/μl)		8500	8300	7300
RBC	(× 10^4^/μl)		618	535	487
Hb	(g/dl)		17.8	15.2	14.9
Hct	(%)		52.9	45.8	44.4
Plt	(× 10^4^/μl)		24.6	22.4	31.6
Renin activity	ng/ml/hr	0.3-2.9	8.8		36.8
Renin concentration	pg/mL	2.5-21.4		53.8	
Urinalysis					
Specific gravity			1.026	1.034	1.028
pH			5.5	5.5	5
Urinaly glucose	(mg/dl)		2366	(4+)	3002
	(g/day)		18.928	N/A	58.5
Protein	(g/day)		0.28	0.06	0.08
Ketone			–	–	–

*BMI* body mass index, *SGLT2i* sodium-glucose transport protein 2 inhibitor, *OHA* oral hyperglycemic agent, *DPP4* Dipeptidyl Peptidase-4, *SU* sulfonylurea, *αGI* α-glucosidase inhibitor, *TP* total protein, *AST* aspartate aminotransferase, *ALT* alanine transaminase, *LDH* lactate dehydrogenase, *ALP* alkaline phosphatase, *γ-GTP* γ-glutamyl transferase, *UN* urea nitrogen, *eGFR* estimated glomerular filtration ratio, *UA* uric acid, *TG* triglyceride, *HDL* high density lipoprotein, *LDL* low density lipoprotein; CRP, C-reactive protein

### Case 1

Male, 41 years old, with a 7-year history of DM, was admitted for evaluation of acute renal dysfunction (from 1.65 mg/dL to 3.0 mg/dL serum creatinine, from 36.7 mL/min/1.73 m^2^ to 19.9 mL/min/1.73 m^2^ eGFR for 56 day intervals). Treatment with dapagliflozin 5 mg daily was started from 19 months before this admission. The patient’s height was 162 cm and weight increased from 84 to 93 kg, following treatment with dapagliflozin. Urinary protein was 0.28 g/day and negative for ketones. The patient’s blood pressure was 128/76 mmHg; HbA1c, 6.1%; blood sugar level, 74 mg/dL; and plasma renin activity was 8.9 ng/ml/hr (normal 0.3–2.9). Renal biopsy was performed to evaluate renal dysfunction.

### Case 2

Male, 55 years old, with a 5-year history of DM, was admitted for evaluation of acute renal dysfunction (from 1.03 mg/dL to 1.21 mg/dL serum creatinine, from 59.5 mL/min/1.73 m^2^ to 50.8 mL/min/1.73 m^2^ eGFR for 56 day intervals). Treatment with dapagliflozin 5 mg daily was started from 24 months before this admission. The patient’s height was 169 cm and weight increased from 88 kg to 95.8 kg following dapagliflozin treatment. Urinary protein was 0.08 g/day and negative for ketones. The patient’s blood pressure was 130/75 mmHg; HbA1c, 6.5%; blood sugar level, 86 mg/dL; and plasma renin concentration, 53.8 pg/mL (normal 2.5–21.4). Renal biopsy was performed to evaluate renal dysfunction.

### Case 3

Male, 52 years old, with a 17-year history of DM, was admitted for evaluation of acute renal dysfunction (from 0.8 mg/dL to 1.1 mg/dL of serum creatinine, 79.7 mL/min/1.73 m^2^ to 68.4 mL/min/1.73 m^2^ of eGFR) for 56 day intervals). Treatment with dapagliflozin 5 mg daily was started from 5 months before this admission. The patient’s height was 164.5 cm and weight increased from 80 to 82 kg following dapagliflozin treatment. Urinary protein was 0.08 g/day, and negative for ketones. The patient’s blood pressure was 122/74 mmHg; HbA1c, 7.4%; blood sugar level, 108 mg/dL; and plasma renin activity was 36.8 ng/ml/hr (normal 0.3–2.9). Renal biopsy was performed to evaluate renal dysfunction.

## Renal histology

### Case 1

Global sclerosis, determined by LM, was observed in 10 out of 42 glomeruli. The preserved glomeruli showed mild mesangial expansion in > 25% of the observed mesangium. Linear staining for immunoglobulin G (IgG) along the glomerular basement membrane (GBM) was observed by IF analysis. The GBM was thickened as revealed by EM analysis with a width of 560–680 nm (> 430 nm). Class IIa DN was diagnosed according to Tervaert’s classification. Arteriolar hyalinosis was severe, and polar vasculosis was observed around the glomeruli. Tubular atrophy and interstitial fibrosis were observed in approximately 15% of the total renal cortex. Considerable amounts of isometric vacuolization were observed on tubules, and was completely negative for Periodic acid-Schiff (PAS) stain.

### Case 2

Global sclerosis was present in 1 out of 22 glomeruli as determined by LM. Preserved glomeruli did not show mesangial expansion. Linear staining for IgG along the GBM was not observed by IF analysis. In addition, EM analysis showed no thickening of the GBM, with a width of 280–380 nm (< 430 nm). This patient did not have class IIa DN according to Tervaert’s classification. Arteriolar hyalinosis was mild. Polar vasculosis was observed around the glomeruli. Tubular atrophy and interstitial fibrosis occupied less than 5% of the total renal cortex. Isometric vacuolization was noted on tubules, and was partially negative for PAS stain.

### Case 3

Global sclerosis was present in 3 out of 32 glomeruli in this patient, as observed via LM analysis. The preserved glomeruli showed mild mesangial expansion > 25% in the mesangium. Linear staining for IgG along the GBM was not observed by IF analysis. The GBM was thickened, as shown by EM analysis, with a width of 450–490 nm (> 430 nm). Arteriolar hyalinosis was mild and polar vasculosis was observed around the glomeruli. Tubular atrophy and interstitial fibrosis were noted in approximately 5% of the total renal cortex. Isometric vacuolization was noted on tubules, and was partially negative for PAS stain.

## Immunohistochemical (IHC) staining for nephron isometric vacuolization of tubules

Immunohistochemistry (IHC) analysis was performed to investigate the isometric vacuolization of tubules as previously described [5]. CD138 and CD10 show proximal convoluted tubule. E-cadherin, EMA and CK 7 show distal tubule. CK34βE12 shows collecting duct (Table 1).

**Fig. 1:**
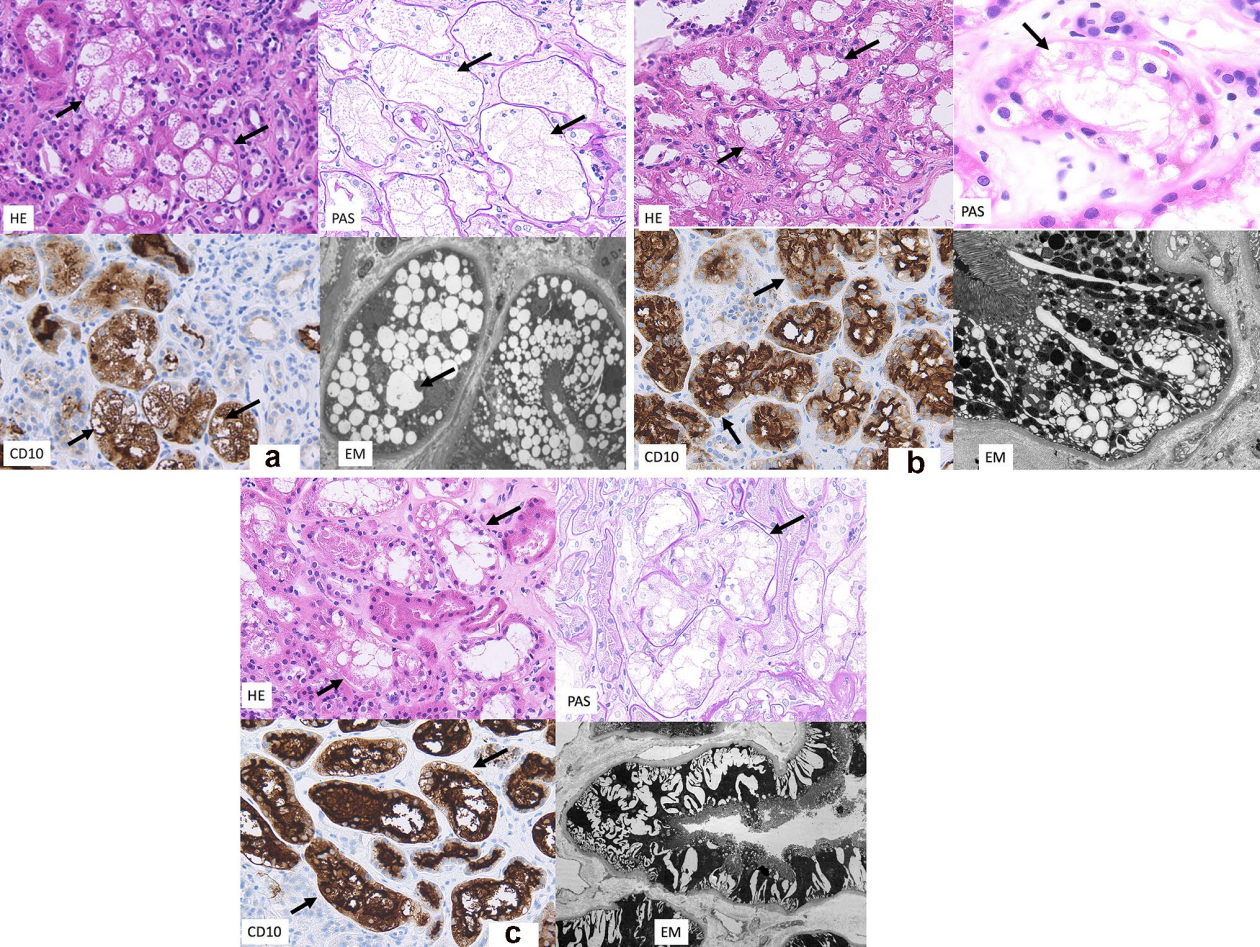
**a** LM analysis of a renal biopsy specimen of case 1 shows that proximal tubule is vacuolated (arrows) (Hematoxylin–Eosin stain, Periodic Acid Schiff stain and CD10 stain 400 ×). Brown color shows positivity for CD 10 stain, consistent with proximal tubule). EM analysis revealed round or elliptical vacuolization as confirmed on proximal tubules. **b** LM analysis of a renal biopsy specimen of case 2 shows that proximal tubule is vacuolated (arrows) (Hematoxylin–Eosin stain, Periodic Acid Schiff stain and CD10 stain 400 ×). Brown color shows positivity for CD 10 stain, consistent with proximal tubule).EM analysis showed round or elliptical vacuolization as confirmed on proximal tubules. **c** LM analysis of a renal biopsy specimen of case 3 showed that proximal tubule is vacuolated (arrows) (Hematoxylin–Eosin stain,, Periodic Acid Schiff stain and CD10 stain 400 ×). Brown color shows positivity for CD 10 stain, consistent with proximal tubule). EM analysis showed curved stripe-formed vacuolization as confirmed on proximal tubules

These three patients showed the following common characteristics; isometric vacuolization of tubules was positive for CD138 and CD10, and negative for E Cadherin, EMA, CK7 and CK34βE2, indicating that isometric vacuolization is localized in proximal convoluted tubules(Fig. 1a–c).

## Electron microscopy (EM) for isometric vacuolization of tubules

### Case 1

Round or elliptical vacuolization was confirmed on proximal tubules by EM analysis (Fig. 1a).

### Case 2

Round or elliptical vacuolization was confirmed on proximal tubules by EM analysis (Fig. 1b).

### Case 3

Curved stripe-formed vacuolization on proximal tubules was confirmed by EM analysis (Fig. 1c).

These large lysosomal vacuoles with some amorphous debris are considered to be due to osmotic nephropathy but convincing evidence of glycogen accumulation was not confirmed.

## Clinical course

Following a strict diet regime, the 3 T2DM patients diagnosed with osmotic vacuolization presented with the following:

### Case 1

Body weight and serum creatinine decreased to 84 kg and 1.6 mg/dL, respectively.

### Case 2

Body weight and serum creatinine decreased to 88 kg and 1.1 mg/dL, respectively.

### Case 3

Body weight and serum creatinine decreased to 80 kg and 0.8 mg/dL, respectively.

Three years after the admission, on these three patients, further deterioration of renal function is not noted with ongoing administration of SGLT2 inhibitor.

## Discussion

The renal pathology of three T2DM cases who suffered AKI after SGLT2 inhibitor treatment was evaluated. Renal biopsy revealed isometric vacuolization of proximal tubules. However, Diabetic and alcoholic ketoacidosis did not exit. All three patients were obese with over 30 of body mass index (BMI). Marked increase in serum renin was definite at this admission, suggesting dehydration. We speculated why SGLT2 inhibitor treatment has a close relation with osmotic vacuolization.

We examined some literature of occurrence of AKI after SGLT2 inhibitor treatment. The US Food and Drug Administration (FDA) has highlighted the risk of AKI following treatment with SGLT2 inhibitors in T2DM patients. A meta-analysis of three clinical trials, comparing the frequency of AKI adverse events in T2DM patients treated with placebo or an SGLT2 inhibitor, reported that SGLT2 inhibitors may protect vulnerable patients with T2DM from AKI [6]. In contrast, a study by Nadkarni showed increased risk of AKI associated with SGLT2 inhibitors in T2DM patients in two large health systems [7]. A study by Donnan et al., however, reported that evidence from randomized control trials (RCTs) does not indicate an increased risk of harm with SGLT2 inhibitors compared with placebo or active comparators, with respect to AKI, DKA, UTI, or fracture. However, this study had wide confidence intervals (CIs) for many comparisons indicating limited precision. Consequently, vital clinical adverse events cannot be ruled out [8].

SGLT2 inhibitors may have nephroprotective effects not only in diabetic patients but also in non-diabetic patients. In addition, SGLT2 inhibitors are more often administered to obese patients because of their weight loss effects via natriuresis with a reduction in plasma volume. While this mechanism has a possibility of easily dehydration [9]. Obesity in our three patients progressed following SGLT2 inhibitor treatment. In addition, severe dehydration might contribute to acute kidney injury, because marked increase in serum renin was definite at this admission. It is well known that SGLT2 inhibitors excrete a large amount of sugar into the urine, resulting in hyperosmolarity of the urine. This does not cause kidney damage in individuals of a healthy weight [9].

Diabetic ketoacidosis (DKA) is characterized by markedly hyperglycemia and acidosis secondary to the elevation of blood ketones resulting from a shortage of insulin. Consequently, an increase in plasma osmolarity, and osmotic diuresis due to high glucose that spill over into the urine, leads to polyuria, and dehydration due to total body water shortage [10]. DKA-related hyperglycemia and subsequent osmotic diuresis have been considered to result in AE lesion of proximal tubule. Typical AE lesion has been described as swelling of proximal tubular cells with cytoplasm of white colored-clear cell change for HE stain due to glycogen accumulation [10]. However, Zhou et al. investigated that vacuolation in proximal tubule could be produced by hyperosmolarity with 20% mannitol at 11 mOsm/kg, and reported that typical PAS-positive clear cell change of cytoplasm could not gained in vacuolated tubule. They emphasized that hyperglycemia and hyperosmolarity is essential for the formation of typical AE lesion, and PAS stain is recommended for the detection of typical AE lesion [11]. While Parai et al. reported that the subnuclear vacuolation of proximal tubule could be noted on patients with non-diabetic alcoholic ketoacidosis, and that this lesion was positive for fat (oil red O) stain, but did not show positivity for PAS stain. Vacuolation in proximal tubule is noted on patients with ketoacidosis of two type; DKA and alcoholic ketoacidosis. Dehydration due to decrease in the fluid volume was a common factor [10–12].

When our three patients were investigated from the view of PAS stain, case 1 was completely negative for PAS stain, but case 2 and 3 were partially negative for PAS stain [10]. Compared among DKD-related AE lesion and our SGLT2-related osmotic vacuolization of proximal tubular epithelial cells, osmotic diuresis due to excretion of large amounts of sugar into urine and dehydration were common factors and might contribute to vacuolization of proximal tubules, although our cases did not show markedly hyperglycemia. There may be relative long periods between the initiation of SGLT2 inhibitor and the onset of AKI on these three patients. This may imply that overeating related to rapid weight gain in a short period of time, and dehydration contribute to AKI clinically, but long-term obesity and dehydration contribute to vacuolization of proximal tubules on chronic phase histologically.

In conclusion, vacuolation of proximal tubular epithelial cells can be induced by various kinds of mechanisms such as ischemia, hypokalemia, hyperosmolarity and lipid accumulation according to the previous reports [10–12]. SGLT2 inhibitor treatment for obese individuals can result in the damage of proximal tubules due to an overdose lavage of sugar into the urine, as well as dehydration related to diuretic hyperosmolarity, resulting in AKI. However, appropriate control of body weight by nutritional diet therapy may prevent the deterioration of renal function even after continuation of SGLT2 treatment in T2DM patients. The current study shows that SGLT2 inhibitor treatment may induce osmotic vacuolization of proximal tubular epithelial cells and AKI for T2DM patients unable to manage a stable weight range.

## Abbreviations

AKI: Acute kidney
CIs: Injury, confidence intervals
CK7: Cytokeratin 7
CK34βE12: Cytokeratin 34-beta E12
DKA: Diabetic ketoacidosis
DM: Diabetes mellitus
DN: Diabetic nephropathy
EM: Electron microscopy
EMA: Epithelial membrane antigen
eGFR: Estimated glomerular filtration rate
FDA: Food and Drug Administration
GBM: Glomerular basement membrane
IF: Immunofluorescence microscopy
IgG: Immunoglobulin G
LM: Light microscopy
SGLT2: Sodium-glucose transport protein 2
T2DM: Type II diabetes mellitus
UTI: Urinary tract infection
